# A vaccine based on the yeast-expressed receptor-binding domain (RBD) elicits broad immune responses against SARS-CoV-2 variants

**DOI:** 10.3389/fimmu.2022.1011484

**Published:** 2022-11-09

**Authors:** Yu Liu, Danhua Zhao, Yichang Wang, Zhian Chen, Li Yang, Wenjuan Li, Yanqiu Gong, Chunmei Gan, Jieshi Tang, Tizhong Zhang, Dan Tang, Xiuju Dong, Qingzhe Yang, C. Alexander Valencia, Lunzhi Dai, Shiqian Qi, Biao Dong, Hoi Yee Chow, Yuhua Li

**Affiliations:** ^1^ National Clinical Research Center for Geriatrics and State Key Laboratory of Biotherapy, West China Hospital, Sichuan University, Chengdu, China; ^2^ Department of Arboviral Vaccine, National Institutes for Food and Drug Control, Beijing, China; ^3^ Department of Urology, Institute of Urology, State Key Laboratory of Biotherapy, West China Hospital, Sichuan University, Chengdu, China; ^4^ College of Life Sciences, Sichuan University, Chengdu, China; ^5^ Sichuan Real & Best Biotech Co., Ltd., Chengdu, China

**Keywords:** SARS-CoV-2, receptor-binding domain, *Pichia pastoris*, Delta, Omicron, vaccine

## Abstract

Development of safe and efficient vaccines is still necessary to deal with the COVID-19 pandemic. Herein, we reported that yeast-expressed recombinant RBD proteins either from wild-type or Delta SARS-CoV-2 were able to elicit immune responses against SARS-CoV-2 and its variants. The wild-type RBD (wtRBD) protein was overexpressed in *Pichia pastoris*, and the purified protein was used as the antigen to immunize mice after formulating an aluminium hydroxide (Alum) adjuvant. Three immunization programs with different intervals were compared. It was found that the immunization with an interval of 28 days exhibited the strongest immune response to SARS-CoV-2 than the one with an interval of 14 or 42 days based on binding antibody and the neutralizing antibody (NAb) analyses. The antisera from the mice immunized with wtRBD were able to neutralize the Beta variant with a similar efficiency but the Delta variant with 2~2.5-fold decreased efficiency. However, more NAbs to the Delta variant were produced when the Delta RBD protein was used to immunize mice. Interestingly, the NAbs may cross react with the Omicron variant. To increase the production of NAbs, the adjuvant combination of Alum and CpG oligonucleotides was used. Compared with the Alum adjuvant alone, the NAbs elicited by the combined adjuvants exhibited an approximate 10-fold increase for the Delta and a more than 53-fold increase for the Omicron variant. This study suggested that yeast-derived Delta RBD is a scalable and an effective vaccine candidate for SARS-CoV-2 and its variants.

## Introduction

The ongoing global COVID-19 pandemic caused by the SARS-CoV-2 virus and its variants poses a major threat to global public health. In response to this epidemic, various types of vaccines have been developed including inactivated SARS-CoV-2 virus, viral vectors, nucleic acids (mRNA and DNA), virus-like particles, and recombinant proteins (1–3). However, the continuous emergence of SARS-CoV-2 variants brought a huge challenge for vaccine development. Several major variants have circulated through the population including Alpha (B.1.1.7), Beta (B.1.351), Gamma (P.1), Delta (B.1.617.2), and Omicron (B.1.1.529). Many vaccines partially or completely lost protective efficacy to these variants, especially to the Delta and Omicron variants (4–6). It was found that, after two-dose vaccination of CoronaVac or BBIBP-CorV, the geometric mean titers (GMTs) of neutralizing antibodies (NAbs) against Delta Variants in plasma decreased 5.1-fold (CoronaVac) or 1.47-fold (BBIBP-CorV) and were below the lower limit for the Omicron variants (6, 7). Similarly, after two doses of vaccination with mRNA-1273, BNT162b2, and AZD1222, NAb titers against Omicron decreased 39-, 37- and 21-fold, respectively (8).

Three recombinant protein vaccines have been licensed as SARS-CoV-2 vaccines (4, 9). Compared with other types of vaccines, recombinant vaccines are highly efficient, secure, and have relatively lower costs. They have been widely used to prevent viral infections caused by Hepatitis B and HPV (10, 11). To produce recombinant proteins, multiple expression platforms including bacteria cells, yeast cells, insect cells, and mammalian cells have been used according to specific requirements (9). Among these different expression systems, the yeast *Pichia pastoris* (*P. pastoris*) system is very attractive for the development of a COVID-19 vaccine. Firstly, methylotrophic cells produce post-translational modifications which is important for the vaccine and which does not happen in *E. coli* (9). Also, this system is less expensive than the insect and mammalian expression systems (12, 13). Furthermore, the extracellular secretion of the antigen, RBD protein, makes the purification process very simple. It has been demonstrated that the yield of SARS-CoV-2 RBD in the *P. pastoris* expression system was higher than in mammalian expression systems (14).

The weak immune responses elicited by the recombinant RBD protein should be overcome if it is used as the COVID-19 vaccine candidate. It has been found that recombinant proteins can only induce weak immune responses in the absence of adjuvants (15, 16). To this end, the selection of adjuvants is the priority for a vaccine based on the RBD protein. Alum is a standard adjuvant that has been used in approved SARS-CoV-2 vaccines such as EpiVacCorona and ZF2001 (4, 17, 18). To further increase the immune responses, unmethylated CpG-oligodeoxynucleotides (CpG) may be used in combination with Alum. CpG can elicit Th1 response *via* toll-like receptor 9 (TLR9), and studies showed that robust humoral immune responses against SARS-CoV-2 were detected in mice immunized with the antigen and a combination of Alum and CpG adjuvants (19–21). Finally, an optimal immunization regimen needs to be investigated. It has been found that the NAb titers against SARS-CoV-2 variants may be significantly increased after a third booster (6, 8).

In this study, to enhance the vaccine potency of the yeast-derived RBD-based recombinant protein vaccine, the immunization regimens and adjuvant selections were compared. Our results showed that either wtRBD or Delta RBD purified from *P. pastoris* cells may be used as an efficient antigen. A three-dose vaccine administration with a 28-day interval was most effective, and higher levels of NAb were induced when Alum and CpG were used as a combined adjuvant.

## Materials and methods

### Plasmid construction

The coding sequence for SARS-CoV-2 RBD (S protein residues 319-541, GenBank: MN908947) was codon-optimized for *P. pastoris* expression. An α-factor signal peptide was added to the N-terminal of RBD for protein secretion and an enterokinase (EK)-cleavable 6×His-tag was added to the C-terminal for purification. The sequence (referred to as wtRBD-co) listed in **Table S1** was synthesized by Anhui Gene Universal Technology Co., Ltd, and the RBD fragment was subcloned into the pPICZ A vector to generate pPICZα A-wtRBD-co.

To construct the plasmid pPICZα A-Delta RBD-co, the SARS-CoV-2 RBD sequence was codon-optimized (referred to as wtRBD-co1) (**Table S1**), synthesized, and inserted into the pUC57 vector by GenScript (Nanjing). This plasmid was used as the template for PCR amplification of wtRBD-co1 using the primer pairs 3/4. The α-factor fragment was obtained with the primer pairs 1/2 using the pPICZα A as the template. The two PCR fragments were joined by overlapping PCR with the primer pairs 1/4 and cloned into the vector pPICZ A *via* EcoRI/SalI double digestions. The resulting plasmid was referred to as pPICZα A-wtRBD-co1. Next, site-specific mutagenesis (L452R and T478K) of the Delta SARS-Cov-2 RBD was performed using primer pairs 1/5 and 4/6, and pPICZα A-wtRBD-co1 served as the template. These two PCR fragments were then joined by overlapping extension PCR with the primer pairs 1/4, and inserted into the vector pPICZ A *via* EcoRI/SalI double digestions to generate the plasmid pPICZα A-Delta RBD-co. The primers used in this study were synthesized by Sangon Biotech (Shanghai) and listed in **Table S2**.

### Cloning of recombinant *P. pastoris*

The plasmids pPICZα A-wtRBD-co and pPICZα A-Delta RBD-co were linearized with SacI and transformed into the *P. pastoris* X33 (Invitrogen, Carlsbad) by electroporation (Bio-Rad, Hercules). Yeast clones integrated with wtRBD-co or Delta RBD-co gene were selected on yeast extract peptone dextrose sorbitol (YPDS) plates containing 100 μg/mL zeocin (Invitrogen) at 30°C. Colony PCR was performed using the primer 5’AOX and 3’AOX (**Table S2**), and the positive clones were selected and grown in 3 mL BMGY medium (Sangon Biotech) at 30°C with a shaking speed of 250 rpm, until the OD600 reached 2.0-6.0. Yeast cells were harvested by centrifugation at 3,000 rpm for 5 min at 4°C, and the pellets were suspended with an equal volume of the BMMY medium (Sangon Biotech). Induction was performed at 30°C with shaking at 250 rpm for 72 h, and additional methanol (0.5% final concentration) was added to the medium every 24 h. The expression levels of RBD protein in the supernatant for individual yeast clones were quantified by western blot analysis.

### Expression and purification of RBD protein

The selected yeast clones were cultured in 300 mL BMGY medium as described above, and the supernatants were harvested at approximately 72 h after induction by centrifugation at 15,000 rpm for 15 min at 4°C. For protein purification, the supernatant containing wtRBD or Delta RBD was filtered with a 0.22 μm filter (Millipore, Billerica) and purified through a 5-mL HisTrap HP column (GE Healthcare, Salt Lake City). Next, the protein was further purified using anion-exchange chromatography (HiTrap Q HP columns) and a size-exclusion column (Superdex 200 Increase 10/300 GL columns, GE Healthcare) (22, 23). The purified protein was digested with Enterokinase (Yeasen, Shanghai) at 4°C overnight to cleave the 6×His-tag. Enterokinase and the tag were removed by a HiTrap Q HP column and a Superdex 200 Increase 10/300 GL column in a buffer containing 20 mM Tris-HCl (pH 8.0) and 150 mM NaCl. The purity and molecular weight of the collected elutions were determined by Coomassie blue staining and western blot analysis. The protein concentration was calculated using a Bradford assay (Thermo Fisher Scientific, Massachusetts).

The samples were denatured by incubating for 10 min at 95°C in a loading buffer containing 1% bromophenol blue, 10% sodium dodecyl sulfate, 50% glycerol, and 60 mmol/L Tris-HCl (pH 6.8) and separated on 10% polyacrylamide gels. Gels were stained with Coomassie blue R-250 to check the purity and quantity. For western blot analysis, the gels were transferred onto PVDF membranes (Millipore) and blocked with TBST buffer containing 5% skimmed milk for 2 h at room temperature. Then, the membranes were incubated with the primary antibody anti-SARS-CoV-2 RBD (1:2000, Sino Biological, Beijing, Cat#40592-T62) overnight at 4°C, followed by the secondary antibody Anti-Rabbit IgG (1:4000, Abbkine, Wuhan) incubation for 1 h at room temperature. Signals were detected using an ECL kit (NCM biotech, Suzhou).

### Analysis of glycosylation sites of wtRBD

The glycosylation sites of wtRBD were analyzed using liquid chromatography-mass spectrometry (LC-MS) (Q Exactive plus mass spectrometer, Thermo Fisher Scientific), and the method has been described previously by Yang et al. (22). Briefly, the purified wtRBD protein was precipitated by adding four volumes of pre-cooled acetone at -20°C overnight and centrifuged at 20,000 g for 10 min at 4°C. The protein precipitate was redissolved in a denaturing buffer (8 M urea, 50 mM NH_4_HCO_3_), and reduced with 20 mM DL-Dithiothreitol (DTT, Sigma, St Louis) for 1 h at 55°C. Furthermore, the proteins were alkylated with 55 mM iodoacetamide (IAA, Sigma) for 30 min at room temperature in the dark, and digested with trypsin at 37°C overnight. After digestion, the peptides were desalted using a C18 ZipTip column according to the manufacturer’s instructions (Millipore), and analyzed by high resolution mass spectrometry using an EASY-nano-LC 1200 (Thermo Fisher Scientific) coupled to a Q-Exactive HF-X (Thermo Fisher Scientific). Raw MS files were further processed using Proteome Discoverer version 2.3 (Thermo Fisher Scientific) with SEQUEST.

### Mouse immunization

All experiments involving mice were approved by the Animal Care and Use Committee of Sichuan University. Female BALB/c mice (6-8 weeks old) were purchased from Ensiweier Biotechnology Co. (Chongqing). After random grouping, mice were intramuscularly immunized with Alum or the mixture of adjuvants (10 µg class C CpG plus 45 µg Alum/mouse) (Parr Bio, Nanjing) and immunogen (wtRBD or Delta RBD) in a total volume of 100 μL, then two further booster immunizations were performed according to the immunization protocols. Blood samples were obtained from retro-orbital venous plexus at 14-day and 28-day after each immunization. The blood samples were placed at room temperature for 2 h, and the sera were collected by centrifugation at 4,000 rpm for 30 min at 4°C. The serum samples were stored at -80°C.

### Measurement of RBD specific antibodies

RBD specific antibodies from the serum samples were measured by ELISA. Briefly, each well of the ELISA plates (Corning Inc, NY) was pre-coated with 200 ng antigens, including wtRBD, Beta RBD, Gamma RBD, Delta RBD or Mink RBD. The wtRBD protein was purchased from Anhui Gene Universal Technology Co., Ltd (Chuzhou), the Beta RBD, Gamma RBD, Y453F RBD protein were obtained from Novoprotein (Shanghai), and the Delta RBD protein was purchased from Beyotime (Shanghai). These antigens were diluted in a coating buffer (35 mM sodium bicarbonate and 15 mM sodium carbonate, pH 9.6), followed by incubation overnight at 4°C. The plates were washed three times with PBST buffer (PBS buffer containing 0.05% Tween-20), and blocked with PBST buffer containing 5% BSA for 1.5 h at room temperature. To detect the binding capacity, serum samples were diluted 100-fold. To measure the RBD-specific titers, serum from immunized mice were serially diluted 3-fold with an initial dilution of 30. The diluted serum samples were added to the plates (100 µL per well), and incubated at room temperature for 1.5 h. After three washes, HRP-conjugated anti-mouse IgG (1:5000, Abbkine, Wuhan) was added (100 µL per well) and incubated for 1.5 h at room temperature. Finally, the plates were detected with 100 µL of 3,3’,5,5’-tetramethyl-biphenyldiamine (TMB) (SeraCare Life Sciences, Milford) for 3 min, and stopped with 1 M HCl solution (100 µL per well). The absorbance values were measured at 450 nm using a microplate reader with a background correction at 630 nm. The serum antibody titers were calculated according to the method described previously by Li et al. (24).

### Neutralization antibody assay

The recombinant Vesicular stomatitis virus (VSV)-based wild-type and variant SARS-CoV-2 pseudoviruses were obtained from the Division of HIV/AIDS and Sex-Transmitted Virus Vaccines, the National Institutes for Food and Drug Control, China. The neutralization assay was carried out as previously published by Nie et al. (25). Briefly, 650 TCID50 of SARS-CoV-2 pseudovirus was mixed with a 3-fold serial dilution of the serum samples starting at 30-fold dilution, followed by incubations at 37°C for 1 h. 2×10^5^ Vero cells were added to each well. After incubating at 37°C with 5% CO_2_ for 24 h, the expression of luciferase was detected using an Ensight plate reader (PerkinElmer, Waltham) for the calculation of NAb titers. The half maximal effective concentration (EC50) of serum samples was calculated. Each plate was set up with a cell-only control (CC) and a virus with cells control (VC). Neutralizing titers below 30 were shown as 30 on the graph.

### Statistical analyses

All data were shown as the mean ± SD and analyzed using GraphPad Prism 8.0. One-way ANOVA with Tukey’s multiple comparisons test was used to analyze the statistical differences among the different groups, and the unpaired Student’s t-test was used to calculate the statistical significance between the two groups. *p*-values lower than 0.05 were considered statistically significant (* *p* < 0.05, ** *p* < 0.01, *** *p* < 0.001, **** *p* < 0.0001) (22).

## Results

### Production of recombinant wtRBD in *P. pastoris*

**Figure 1:**
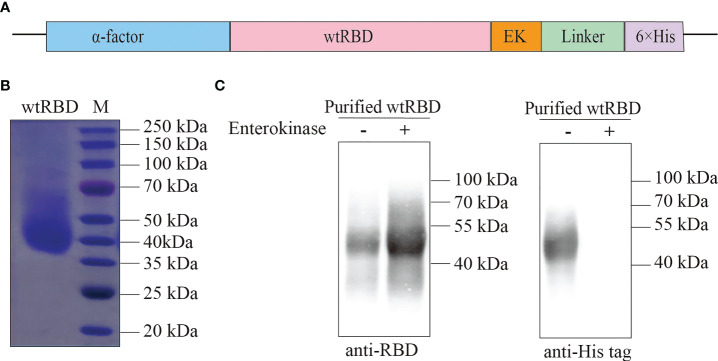
Purification of recombinant wtRBD protein from *Pichia pastoris*. **(A)** Schematic of the expression vector for the recombinant wtRBD protein expressed in yeast. wtRBD contained the α-factor signal peptide (blue) at the N terminus, and an Enterokinase (EK) cleavage site (orange) and a 6×His-tag (purple) at the C terminus. **(B)** The purity analysis of the purified recombinant wtRBD by Commassie blue staining. **(C)** Western blot analysis using SARS-CoV-2 RBD antibody (left) and 6×His antibody (right).

Yeast has been shown to be an ideal system for the large-scale production of recombinant proteins (13, 14); thus, it was used in this study. The vector to overexpress wtRBD was shown in **Figure 1A**. The α-factor signal peptide, a yeast signal peptide, was fused to the N terminus of wtRBD for secretion, and an EK-cleavable 6×His tag was added to the C terminus for purification. The wtRBD was purified as described in the Materials and Methods, and the purity was revealed by SDS PAGE (**Figure 1B**), and the identity was further confirmed by western blot analysis with anti-RBD (SARS-CoV-2) and anti-His antibodies (**Figure 1C**). The purified wtRBD with or without EK digestion was detected using anti-RBD antibody while His-tag was not detected after EK digestion, suggesting nearly all of the protein tags were removed. In contrast to the predicted molecular weight of ~26 kDa, the purified wtRBD was found to be around 45 kDa, suggesting that the purified wtRBD was highly glycosylated (**Figure 1C**). Thus, the purified wtRBD after EK digestion was used to analyze the glycosylation sites using mass spectrometry (MS). As shown in **Figure S1A**, O-glycosylation sites and N-glycosylation sites were identified by Proteome Discoverer, and the presence of these sites have been proven not to affect the binding or recognition with ACE2 receptor (22). The glycosylation of the yeast-derived RBD proteins was further confirmed by PNGase F digestion (**Figures S1B, C**). Together, it was shown that wtRBD was produced efficiently using the *P. pastoris* expression system.

### Immune responses against SARS-CoV-2 elicited by yeast-expressed wtRBD

**Figure 2:**
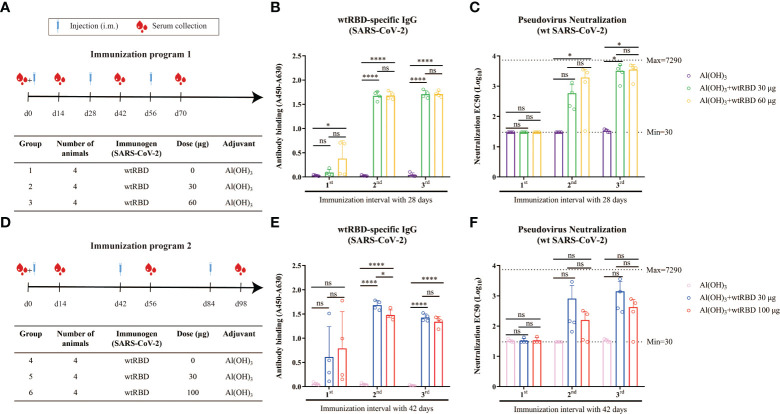
Humoral immune responses induced by yeast-expressed wtRBD in mice. Mice were intramuscularly immunized with different amounts of wtRBD with Alum adjuvant in two different immunization programs. The serum samples were collected at the fourteen day after each immunization and 1^st^, 2^nd^, 3^rd^ indicated the serum samples collected the fourteen day after the first, second, and third immunization, respectively. The serum samples were diluted at a ratio of 1:100 for ELISA to detect IgG binding antibodies. NAbs were quantified based on a pseudovirus assay. **(A)** Schematic of immunization program 1. Each mouse received different amounts of wtRBD three times with a 28-day interval. **(B)** Detection of the wtRBD-specific binding IgG antibodies in serum according to immunization program 1. **(C)** Quantification of NAbs in serum according to immunization program 1. **(D)** Schematic of immunization program 2. Each mouse received different amounts of wtRBD three times at 42-day intervals. **(E)** Detection of the wtRBD-specific binding IgG antibodies in serum according to immunization program 2. **(F)** Quantification of NAbs in serum according to immunization program 2. Data are the mean ± SD. *p*-values were calculated using one-way ANOVA. *p < 0.05, ****p < 0.0001; ns, not significant.

To evaluate the protective efficacy of the yeast-expressed wtRBD as a vaccine, mice were randomized into three groups according to immunization program 1 (**Figure 2A**). Each mouse received 0, 30, or 60 μg of wtRBD by intramuscular immunization for three times 28 days apart, and Alum was used as an adjuvant. Serum samples were collected 14 days after each immunization, and wtRBD-specific IgG and NAbs were detected. As expected, humoral immune responses were induced in both groups immunized with wtRBD antigens, and the dosage effect was observed after the first immunization according to evaluation of absorbance values (**Figure 2B**). Additionally, both wtRBD-specific IgG antibodies and NAbs significantly increased after the second and third immunizations (**Figures 2B, C**). The amounts of these two types of antibodies were also similar after the second and third immunizations according to immunization program 1. Notably, there was no statistical difference in the antibodies between the two groups immunized with 30 μg and 60 μg of wtRBD protein, respectively, indicating that 30 μg of wtRBD was enough as an antigen in mice.

Another immunization program was also set up to evaluate the potency of yeast-derived wtRBD in which each mouse was immunized with 0, 30, or 100 μg of protein three times 42 days apart (**Figure 2D**). Consistent with our results from immunization program 1, robust humoral immune responses were elicited after the second and third immunizations, suggesting that booster injections were necessary (**Figures 2E, F**). It was also found that there was no significant difference in antibody production between the two groups using 30 μg or 100 μg of protein as antigen. In conclusion, these data demonstrated that yeast-expressed wtRBD as antigen can elicit a strong protective immune response, and 30 μg of wtRBD plus Alum adjuvant was sufficient.

### Cross-reactivity against SARS-CoV-2 variants by the vaccine using yeast-expressed wtRBD in mice

**Figure 3:**
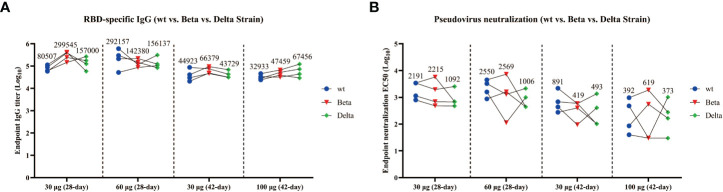
Cross-reactivity of the antisera from mice immunized with wtRBD against Beta and Delta SARS-CoV-2 variants. The endpoint serum samples from mice immunized with wtRBD according to immunization program 1 and 2, which had the immunization intervals of 28 and 42 days, respectively, were used for the IgG binding antibody and NAb assay. **(A)** Titers for IgG binding antibody against the Beta and Delta variant. **(B)** Titers for NAbs against the Beta and Delta variant. Each symbol indicates data from one mouse (n = 4 mice/group); geometric means of each group are shown.

To analyze whether the antisera from the mice immunized with wtRBD at different time intervals can efficiently prevent the SARS-CoV-2 variants, the total IgG binding antibodies and NAbs in the endpoint serum samples were detected. As shown in **Figure 3A**, wtRBD induced the IgG antibodies bound the wild-type, Beta (B.1.351) and Delta (B.1.617.2) variants with similar efficiency, regardless of the 28-day or 42-day immunization program used in this study. This result was further supported by the IgG binding assays for the Gamma (P.1) and Mink (Y453F) variants (**Figure S2**). Notably, relative more binding antibodies were elicited in mice using the program 1 compared with using the program 2 (**Figure S3**).

In addition, compared with the NAb titers against wild-type SARS-CoV-2, no significant difference was found for Beta variant with the 28-day immunization program (**Figure 3B**). However, the NAb titers against the Delta variant were reduced by 2.0 folds and 2.5 folds in the groups immunized with 30 and 60 μg of wtRBD, respectively (**Figure 3B**). Interestingly, the NAb titers obtained by the program 1 were still higher than those by the program 2, further suggesting that a 28-day immunization regimen was superior to a 42-day regimen. Overall, cross-reactive NAbs against SARS-CoV-2 variants were elicited by the yeast-derived wtRBD vaccine in mice. Our results also showed that the Delta variants had greater vaccine escape capacity than the wild-type and Beta SARS-CoV-2, which was consistent with the results from the previous studies (4, 26, 27).

### Immune response induced by yeast-expressed Delta RBD protein in mice

**Figure 4:**
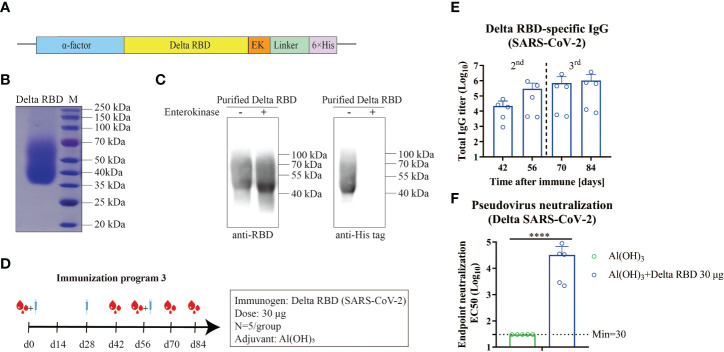
Humoral immune responses elicited by yeast-expressed Delta RBD in mice. **(A)** The schematic of the expression vector for Delta RBD in yeast. Delta RBD contained the α-factor signal peptide (blue) at the N terminus, and an Enterokinase (EK) site (orange) and a 6×His-tag (purple) at the C terminus. **(B)** The purity analysis of the purified recombinant Delta RBD stained by Commassie blue staining. **(C)** The identity of Delta RBD characterized by western blot analysis with the SARS-CoV-2 RBD antibody (left) and 6×His antibody (right). **(D)** Schematic of immunization program 3. Each mouse received 30 μg of Delta RBD protein for three times with an interval of 28-days. Serum samples were collected at the fourteen day after each immunization. **(E)** Measurement of the Delta RBD-specific binding IgG antibodies in the serum. **(F)** Quantification of NAbs against the Delta variant in the endpoint (day 84) serum. Data are the mean ± SD. *p*-values were calculated using unpaired Student’s t-test. ****p < 0.0001.

Given the immune evasion of the Delta variant, the vaccine effectiveness of the Delta RBD protein was investigated. The yeast-expressed Delta RBD protein was overexpressed and purified in a similar approach as described above for wtRBD (**Figure 4A**). The purity and identity were revealed by Coomassie staining (**Figure 4B**) and western blot analysis (**Figure 4C**).

To assess the immunogenicity of the yeast-derived Delta RBD, mice were immunized with 30 μg of the Delta RBD in the presence of Alum according to the immunization program 3 (**Figure 4D**). The Delta RBD-binding antibody significantly increased after the second immunization, and further increased after the third injection (**Figure 4E** and **Figure S4**). At the study endpoint (day 84), the GMTs of Delta RBD-specific IgG in sera were 1028641 (7844–3720491), which was similar to that quantified using wtRBD (**Figure S5**). However, the GMTs of NAb against the Delta variant elicited by the Delta RBD in the endpoint serum was 32255 (2167–88084) (**Figure 4F**). This GMT represented a 5.2-fold increase compared with wtRBD, which was 6202 (899–16199) (**Figure S5**). Altogether, these results indicated that the yeast-derived Delta RBD was more effective in protecting mice from the Delta infection using immunization program 3.

### Immune responses enhanced by CpG+Alum adjuvant with Delta RBD

**Figure 5:**
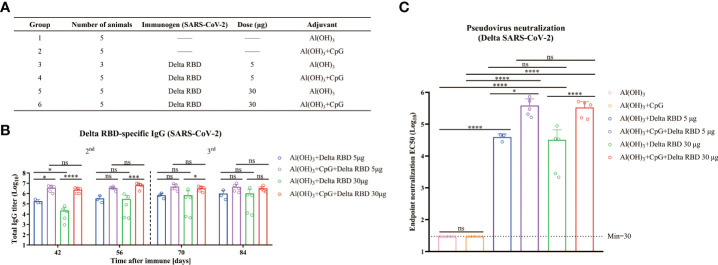
Humoral immune responses enhanced by formulating Delta RBD in conjunction with CpG+Alum adjuvant. **(A)** The treatment for different groups of mice. Mice were randomly grouped and intramuscularly injected according to immunization program 3. **(B)** Measurement of Delta RBD-specific IgG titers in serum. **(C)** Quantification of the NAb titers against the Delta variant in the endpoint serum samples. Data are the mean ± SD. *p*-values were calculated using one-way ANOVA. *p < 0.05, ***p < 0.001, ****p < 0.0001; ns, not significant.

Knowing that immunization with a third dose of many vaccines improves the neutralization breadth (6–8), we hypothesized that higher NAb titers would enhance the vaccine cross-reactivity to different emerging variants. To this end, a combined adjuvant (CpG+Alum) was investigated. The mice were grouped as listed in **Figure 5A** and various adjuvants with Delta RBD were immunized according to immunization program 3. It was observed that for both 5 μg and 30 μg antigen groups, the levels of the Delta RBD-binding antibodies were significantly higher in the groups immunized with CpG+Alum adjuvant than the groups immunized with Alum adjuvant alone after the second immunization (**Figure 5B**). Meanwhile, when CpG adjuvant was combined with Alum, the group with 5 μg of antigen achieved similar amounts of binding antibody as the group with 30 μg of antigen (**Figure 5B**).

To detect the NAbs for the Delta RBD, the endpoint serum samples were used. It was demonstrated that the GMTs of NAb with the CpG+Alum adjuvant induced 9.8-fold higher titers than the Alum adjuvant alone (385546 vs 39494) in the presence of 5 μg of Delta RBD, and it was 10.4-fold higher when 30 μg of Delta RBD was used (335614 vs 32255) (**Figure 5C**). This result suggested that the CpG+Alum adjuvant enhanced the production of NAbs. It was further supported by the results from IgG antibodies and NAbs against the Delta SARS-CoV-2 variant in sera from mice immunized with wtRBD plus CpG+Alum or Alum alone (**Figure S6**). It was observed that there was no significant difference in the amounts of NAbs between 5 μg and 30 μg antigen doses after the third immunization. Collectively, our data suggested that the immunogenicity raised by the yeast-derived Delta RBD was remarkably enhanced by the CpG+Alum adjuvant combination.

### More neutralizing antibodies against Omicron variants elicited by the CpG+Alum adjuvant with Delta RBD

Recently, the Omicron variant has attracted the most attention for its enhanced infectivity and transmissibility (28). Ai et al. and Zhang et al. also showed that Omicron has a significant immune evasion compared with other SARS-CoV-2 variants (6, 29). Therefore, we inquired whether the antisera from the immunized mice contained IgG and NAbs against Omicron variants.

**Figure 6:**
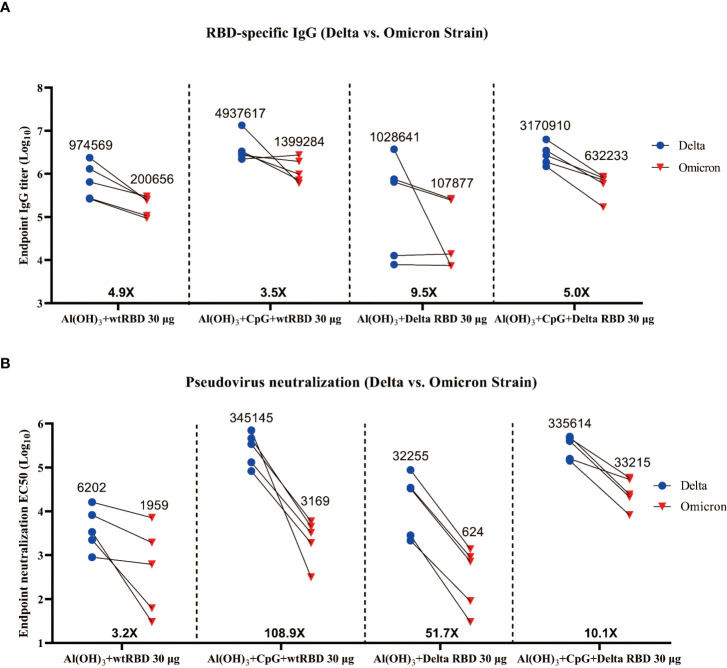
Cross-reactivity of the antisera from mice immunized with wtRBD or Delta RBD against the Omicron variant. The endpoint (day 84) serum samples from the mice immunized with 30 μg Delta RBD (**Figure 5A**) or wtRBD (**Figure S6**) were used for the IgG binding antibody and NAb assays. **(A)** Measurement of the titers of the Omicron RBD-specific IgG antibodies. **(B)** Measurement of the NAb titers against Omicron. Each symbol indicates data from one mouse (n = 5 mice/group); geometric means of each group are shown.

In the binding antibody assay, all of the endpoint (day 84) serum samples from mice immunized with 30 μg of the Delta RBD and adjuvants exhibited a 5.0- to 9.5-fold reduction in RBD-specific IgG titers against Omicron (**Figure 6A**). It was demonstrated that the decrease was even more pronounced when the Alum adjuvant was utilized alone. A similar trend was seen in the 5 μg group (**Figure S7**). The NAbs against Omicron showed a 10.1- to 51.7-fold decrease in the antisera compared with the ones against the Delta variant (**Figure 6B**). For the “Al(OH)_3_+CpG+Delta RBD 30 μg” group, the GMTs of NAbs against Omicron was 54 times higher compared with the “Al(OH)_3_+Delta RBD 30 μg” group (33215 vs 614), indicating that the combination of Delta RBD and CpG+Alum adjuvants was more effective to elicit a protective immune response against Omicron.

Similarly, endpoint (day 84) antisera from mice immunized with 30 μg of wtRBD in conjunction with Alum or CpG+Alum also showed a reduction in IgG and NAbs against Omicron (**Figure S8**). Interestingly, in the presence of CpG+Alum, no significant differences were observed in the GMTs of IgG (3170910 vs 4937617) (**Figure 6A**) and NAbs (335614 vs 345145) (**Figure 6B**) against the Delta variant between 30 μg of Delta RBD and wtRBD, but a 10.5-fold decrease in NAbs against Omicron (33215 vs 3169) was observed (**Figure 6B**). This suggested that the wtRBD has less cross-protection against Omicron. In summary, antisera stimulated by the yeast-derived Delta RBD with CpG+Alum adjuvant was effective against Omicron, which demonstrated its broad-spectrum neutralizing ability to prevent SARS-CoV-2 variants.

## Discussion

RBD is one of the major target antigens for COVID-19 vaccines. It is part of the S1 subunit of the spike and plays an important role in interacting with the ACE2 receptor (30, 31). The safety and efficacy of RBD-based vaccines have been proven in clinical trials (32). However, a more cost-effective and broad-spectrum vaccine is needed to protect against SARS-CoV-2 and its variants. In this study, we systematically explored the effectiveness of a yeast-derived recombinant RBD as a vaccine candidate.

Although various COVID-19 vaccines have been used worldwide and the efficiencies are very encouraging, the cost and storage conditions are still challenging. There is an urgent need for a low-cost and efficient COVID-19 vaccine, such as a yeast-derived Delta RBD, for developing countries. The latest world data showed that 64.3% of people globally have received at least one dose of the COVID-19 vaccine, but this number is only 14.5% in low-income countries (33). This may be one of the reasons why the Beta, Delta, and Omicron variants have caused three epidemic peaks in South Africa (34).

In this study, it was demonstrated that RBD proteins, either wtRBD or Delta RBD, were efficiently overexpressed and purified from yeast *P. pastoris*. Impressively, the final yield was around 4 mg/L for each protein. The production of RBD may be increased markedly in a bioreactor since the yield of wtRBD in a bioreactor can be up to 45 mg/L (14). Several studies demonstrated that yeast is a more scalable and cost-effective expression system than mammalian cells for the production of SARS-CoV-2 RBD (14, 30, 35). To purify the secreted RBD protein in media, a His-tag was fused to the protein. However, few attention has been paid to the elimination of the tag during purification, which may cause safety concerns in the clinical application. To overcome this limitation, an efficient and specific enterokinase (EK) cleavage site was added between the RBD and the His-tag. As expected, the tag was successfully removed from the purified protein (**Figures 1C**, **4C**). In a binding antibody assay using the serum samples raised by CHO cell-derived RBD (36), it was also noted that the antigenicity of RBD protein derived from yeast cells was similar to that derived from mammalian cells (**Figure S9**).

Due to the low immunogenicity of recombinant proteins-based vaccines, multiple immunizations are generally required (9). We therefore explored whether the vaccine efficiency may be affected by the intervals between immunizations. In the present study, immunizations with wtRBD were performed three times at 28-day or 42-day intervals. Compared with the 42-day interval, the IgG antibody titer exhibited a 1.8-fold increase in the 28-day interval (44923 vs 80507) (**Figure 3A**). Similarly, the GMTs of NAb against wild-type SARS-CoV-2 after 3-dose immunizations with a 28-day interval was 2.5-fold higher than that with a 42-day interval (2214 vs 891) (**Figure 3C**). Moreover, after a 3-dose vaccination with Delta RBD, NAbs against the Delta SARS-CoV-2 also showed a 13-fold increase in the immunization program with a 28-day interval compared to that with a 14-day interval (**Figure S10**). Altogether, it was demonstrated that a 28-day interval between immunizations elicited more protective immune responses than a 14-day or 42-day intervals when using the yeast-derived RBD antigen, providing a reliable reference for future vaccine development.

The main challenge now for SARS-CoV-2 prevention is the continuous emergence of new variants rapidly, especially those listed in the VOC (5, 29, 32), that reduced the effectiveness of many vaccines. Although there was some cross-reactivity to Beta and Delta, the Omicron was barely detectable (6, 7). Our study found an obvious decrease of Delta NAb titers in antisera from mice immunized with wtRBD vaccines, which was consistent with previous studies (37, 38). To address this issue, yeast-derived Delta RBD was overexpressed and purified as an antigen. As expected, a strong humoral immune response against the Delta variant was found in mice immunized with Delta RBD and Alum adjuvant (**Figure 4**).

To further enhance the immunogenicity of yeast-derived Delta RBD proteins, a combination of the Th1 adjuvant class C CpG and the Th2 adjuvant Alum were utilized. Our result demonstrated that significantly higher NAb titers against the Delta variant were induced in mice immunized with this combination of antigen and double adjuvants compared with the same antigen plus Alum adjuvant alone (**Figure 5C**). The increases were approximately 10-fold for both 5 μg and 30 μg doses. These results were consistent with other studies (19, 20, 35). Notably, in the 30 μg of yeast-derived Delta RBD group, the GMT of IgG was 3170910 (range from 1483840 to 6297514), and the titer of NAb was 335614 (range from 143460 to 508024). Such high NAbs may be one of the important reasons why the omicron variant was effectively neutralized in this study (**Figure 6B**). In addition, no significant difference in the induction of the protective immune responses against Delta variants were observed between 5 μg and 30 μg of Delta RBD antigen treatments, suggesting that a plateau of protective immune responses may be reached using 5 μg of Delta RBD (**Figures 5B, C**). Such a low amount of antigen showed a great potential for Delta RBD to be applied as a vaccine candidate against Delta SARS-CoV-2 variants.

For the currently circulating Omicron variant (B.1.1.529), a similar NAbs trend was observed (**Figure 6B**). It was found that higher immune responses were elicited when Alum and CpG were used as a combined adjuvant regardless of Delta RBD or wtRBD as immunogen. In the absence of CpG, the GMT of NAbs against Omicron variant dropped to 1959 and 624 at a dose of 30 μg of wtRBD or Delta RBD, respectively (**Figure 6B**). However, in the presence of Alum and CpG, the GMT of NAbs against Omicron variant dropped only to 3169 and 33215, respectively. Toll-like receptor agonists (CpG) can directly activate the Th1-biased immune responses, stimulate plasmacytoid DC and B cells, and secrete the IL-6 and IFN-α, which induce more efficient immune responses. And it was seen that the NAb titers to Omicron was greatly reduced in the antiserum and it still remained a high titer at 30 μg of Delta RBD (**Figure 6B**), which was significantly higher than that of wtRBD-induced NAb (33215 vs 3169) (**Figure 6B**), implying that the Delta RBD vaccine candidate could be better against SARS-CoV-2 variants than the wtRBD vaccine candidate. This could be caused by the Delta and Omicron variants share the same mutation site T478K, which increases the binding affinity to ACE2 (39, 40). Thus, our work fully demonstrated the broad-spectrum capability and potential of yeast-derived Delta RBD as a vaccine candidate, making it possible to potentially prevent more SARS-CoV-2 variants.

Additionally, the recombinant RBD-based vaccine platforms have shown excellent safety and lower cost compared with the mRNA and inactivated vaccine platforms, and have been used for the prevention of HB and HPV. mRNA is one type of novel vaccine and showed great potency. However, it has never been applied for human vaccine production before COVID-19, so its long-term efficiency and safety, especially for myocarditis (41), need to be further evaluated. Traditional inactivated vaccine can be produced in mature platforms, but the high costs and complicated processes limit its access to vaccination in many middle-income and low-income countries. In this study, low intrinsic immunogenicity of yeast-derived RBD-based vaccine candidates was made up by the incorporation of a novel CpG adjuvant to a traditional Alum adjuvant, inducing highly protective immune responses against SARS-CoV-2 and its variants.

The evidence from clinical trials for malaria vaccine MSP1 42-C1 and COVID-19 vaccine SCB-2019 suggested that the CpG was a safe and potent adjuvant and could be further used in the next generation of vaccines (42, 43). It has also been demonstrated that more NAbs could be raised using the combination of Alum and CpG adjuvants in many studies (19, 20, 44), which was consistent with our findings (**Figures 5B, C**). However, such a vaccine would cost a bit more considering the CpG price, which should be noticed especially for the people in middle- and low-income countries.

One of the limitations of this study was the detection of antibodies in a relatively short period. The expression levels of RBD-binding antibodies and NAbs were measured only at week 4 after the third immunization. However, Zang et al. have demonstrated that the mice treated with yeast-derived immunogens remained to protect against the SARS-CoV-2 challenge at week 18 after the third immunization (35), suggesting that the yeast-derived RBD-based recombinant protein vaccine may induce a long-lasting immune response. Another limitation was the small number of mice was used, which decreased the ability to calculate the differences between the immunized and control groups.

In summary, the *P. pastoris*-expressed Delta RBD protein in conjunction with CpG+Alum adjuvant was able to efficiently induce NAbs against SARS-CoV-2 and its variants, which hold great potential as a low-cost and efficient vaccine candidate.

## Data availability statement

The original contributions presented in the study are included in the article/**Supplementary Material**. Further inquiries can be directed to the corresponding authors.

## Ethics statement

The animal study was reviewed and approved by the Ethics Committee of Sichuan University.

## Author contributions

YLi, HC and BD designed the study and oversaw the scientific direction. YLiu analysed data and drafted the manuscript. YLi, HC, BD, SQ, CV, and JT revised the manuscript. YLiu, ZC, YW, and QY constructed the plasmids and recombinant yeast strains. YLiu, ZC, LY, and CG performed the animal experiments. YLi, DZ, and WL performed the pseudovirus neutralization tests. SQ, YW, TZ, DT, and XD purified the recombinant proteins. YG and LD analysed the glycosylation sites of the wtRBD protein. All authors contributed to the article and approved the submitted version.

## Funding

This work was supported by the Scientific and Technological Research Project for Novel Coronavirus Pneumonia, West China Hospital, Sichuan University (HX2019nCoV054) to BD. The National Clinical Research Centre for Geriatrics, West China Hospital, Sichuan University (Z20191002) to BD. Engineering, Medical and Agricultural Development Cooperation Project (00402055A1005) to LD. The authors declare that this study received funding from Sichuan Real & Best Biotech Co., Ltd, (Z0H0362) to SQ, and (Z0H0360) to LD. The funder was not involved in the study design, collection, analysis, interpretation of data, the writing of this article, or the decision to submit it for publication.

## Acknowledgments

We thank Division of HIV/AIDS and Sex-Transmitted Virus Vaccines, the National Institutes for Food and Drug Control for providing the recombinant Vesicular stomatitis virus (VSV)-based wild-type and variant SARS-CoV-2 pseudoviruses.

## Conflict of interest

Author BD is employed by Sichuan Real & Best Biotech Co., Ltd., Chengdu, China.

The remaining authors declare that the research was conducted in the absence of any commercial or financial relationships that could be construed as a potential conflict of interest.

## Publisher’s note

All claims expressed in this article are solely those of the authors and do not necessarily represent those of their affiliated organizations, or those of the publisher, the editors and the reviewers. Any product that may be evaluated in this article, or claim that may be made by its manufacturer, is not guaranteed or endorsed by the publisher.

## References

[1] Ita K. Coronavirus disease (COVID-19): Current status and prospects for drug and vaccine development. Arch Med Res (2021) 52(1):15–24. doi: 10.1016/j.arcmed.2020.09.010 32950264 PMC7832760

[2] Awadasseid A Wu Y Tanaka Y Zhang W. Current advances in the development of SARS-CoV-2 vaccines. Int J Biol Sci (2021) 17(1):8–19. doi: 10.7150/ijbs.52569 33390829 PMC7757035

[3] Yadav T Srivastava N Mishra G Dhama K Kumar S Puri B. Recombinant vaccines for COVID-19. Hum Vaccin Immunother (2020) 16(12):2905–12. doi: 10.1080/21645515.2020.1820808 PMC7711739 33232211

[4] Fiolet T Kherabi Y MacDonald CJ Ghosn J Peiffer-Smadja N. Comparing COVID-19 vaccines for their characteristics, efficacy and effectiveness against SARS-CoV-2 and variants of concern: a narrative review. Clin Microbiol Infect (2022) 28(2):202–21. doi: 10.1016/j.cmi.2021.10.005 PMC8548286 34715347

[5] Garcia-Beltran WF Lam EC St Denis K Nitido AD Garcia ZH Hauser BM. Multiple SARS-CoV-2 variants escape neutralization by vaccine-induced humoral immunity. Cell (2021) 184(9):2372–2383.e9. doi: 10.1016/j.cell.2021.03.013 33743213 PMC7953441

[6] Ai J Zhang H Zhang Y Lin K Zhang Y Wu J. Omicron variant showed lower neutralizing sensitivity than other SARS-CoV-2 variants to immune sera elicited by vaccines after boost. Emerg Microbes Infect (2022) 11(1):337–43. doi: 10.1080/22221751.2021.2022440 PMC8788341 34935594

[7] Wang K Jia Z Bao L Wang L Cao L Chi H. Memory b cell repertoire from triple vaccinees against diverse SARS-CoV-2 variants. Nature (2022) 603(7903):919–25. doi: 10.1038/s41586-022-04466-x PMC8967717 35090164

[8] Cameroni E Bowen JE Rosen LE Saliba C Zepeda SK Culap K. Broadly neutralizing antibodies overcome SARS-CoV-2 omicron antigenic shift. Nature (2022) 602(7898):664–70. doi: 10.1038/s41586-021-04386-2 PMC9531318 35016195

[9] Pollet J Chen WH Strych U. Recombinant protein vaccines, a proven approach against coronavirus pandemics. Adv Drug Delivery Rev (2021) 170:71–82. doi: 10.1016/j.addr.2021.01.001 PMC7788321 33421475

[10] Michel ML Tiollais P. Hepatitis b vaccines: protective efficacy and therapeutic potential. Pathol Biol (Paris) (2010) 58(4):288–95. doi: 10.1016/j.patbio.2010.01.006 20382485

[11] Schmiedeskamp MR Kockler DR. Human papillomavirus vaccines. Ann Pharmacother (2006) 40(7-8):1344–52. doi: 10.1345/aph.1G723 16849621

[12] Ahmad M Hirz M Pichler H Schwab H. Protein expression in *Pichia pastoris*: recent achievements and perspectives for heterologous protein production. Appl Microbiol Biotechnol (2014) 98(12):5301–17. doi: 10.1007/s00253-014-5732-5 PMC4047484 24743983

[13] Karbalaei M Rezaee SA Farsiani H. *Pichia pastoris*: A highly successful expression system for optimal synthesis of heterologous proteins. J Cell Physiol (2020) 235(9):5867–81. doi: 10.1002/jcp.29583 PMC7228273 32057111

[14] Consortium AA. Structural and functional comparison of SARS-CoV-2-spike receptor binding domain produced in *Pichia pastoris* and mammalian cells. Sci Rep (2020) 10(1):21779. doi: 10.1038/s41598-020-78711-6 33311634 PMC7732851

[15] Brito LA O'Hagan DT. Designing and building the next generation of improved vaccine adjuvants. J Control Release (2014) 190:563–79. doi: 10.1016/j.jconrel.2014.06.027 24998942

[16] Skwarczynski M Toth I. Micro and nanotechnology in vaccine development: Nanomaterials based on lipids for vaccine development. New York (NY: Elsevier William Andrew (2017). doi: 10.1016/B978-0-323-39981-4.00013-0

[17] He P Zou Y Hu Z. Advances in aluminum hydroxide-based adjuvant research and its mechanism. Hum Vaccin Immunother (2015) 11(2):477–88. doi: 10.1080/21645515.2014.1004026 PMC4514166 25692535

[18] Liang Z Zhu H Wang X Jing B Li Z Xia X. Adjuvants for coronavirus vaccines. Front Immunol (2020) 11:589833. doi: 10.3389/fimmu.2020.589833 33240278 PMC7677582

[19] Kuo TY MY L Coffman RL Campbell JD Traquina P Lin YJ. Development of CpG-adjuvanted stable prefusion SARS-CoV-2 spike antigen as a subunit vaccine against COVID-19. Sci Rep (2020) 10(1):20085. doi: 10.1038/s41598-020-77077-z 33208827 PMC7676267

[20] Nanishi E Borriello F O'Meara TR McGrath ME Saito Y Haupt RE. Alum:CpG adjuvant enables SARS-CoV-2 RBD-induced protection in aged mice and synergistic activation of human elder type 1 immunity. bioRxiv (2021). doi: 10.1101/2021.05.20.444848

[21] Liang JG Su D Song TZ Zeng Y Huang W Wu J. S-trimer, a COVID-19 subunit vaccine candidate, induces protective immunity in nonhuman primates. Nat Commun (2021) 12(1):1346. doi: 10.1038/s41467-021-21634-1 33649323 PMC7921634

[22] Yang J Wang W Chen Z Lu S Yang F Bi Z. A vaccine targeting the RBD of the s protein of SARS-CoV-2 induces protective immunity. Nature (2020) 586(7830):572–7. doi: 10.1038/s41586-020-2599-8 32726802

[23] Dai L Zheng T Xu K Han Y Xu L Huang E. A universal design of betacoronavirus vaccines against COVID-19, MERS, and SARS. Cell (2020) 182(3):722–733.e11. doi: 10.1016/j.cell.2020.06.035 32645327 PMC7321023

[24] Li W Li X Zhao D Liu J Wang L Li M. Heterologous prime-boost with AdC68- and mRNA-based COVID-19 vaccines elicit potent immune responses in mice. Signal Transduct Target Ther (2021) 6(1):419. doi: 10.1038/s41392-021-00843-6 34903732 PMC8666615

[25] Nie J Li Q Wu J Zhao C Hao H Liu H. Quantification of SARS-CoV-2 neutralizing antibody by a pseudotyped virus-based assay. Nat Protoc (2020) 15(11):3699–715. doi: 10.1038/s41596-020-0394-5 32978602

[26] Harvey WT Carabelli AM Jackson B Gupta RK Thomson EC Harrison EM. SARS-CoV-2 variants, spike mutations and immune escape. Nat Rev Microbiol (2021) 19(7):409–24. doi: 10.1038/s41579-021-00573-0 PMC8167834 34075212

[27] Raman R Patel KJ Ranjan K. COVID-19: Unmasking emerging SARS-CoV-2 variants, vaccines and therapeutic strategies. Biomolecules (2021) 11(7):993. doi: 10.3390/biom11070993 34356617 PMC8301790

[28] Callaway E. Heavily mutated omicron variant puts scientists on alert. Nature (2021) 600:21. doi: 10.1038/d41586-021-03552-w 34824381

[29] Zhang L Li Q Liang Z Li T Liu S Cui Q. The significant immune escape of pseudotyped SARS-CoV-2 variant omicron. Emerg Microbes Infect (2022) 11(1):1–5. doi: 10.1080/22221751.2021.2017757 PMC8725892 34890524

[30] Maria P Talha A Susan PR Venkata VE Katharine F Justin CS. A yeast expressed RBD-based SARS-CoV-2 vaccine formulated with 3M-052-alum adjuvant promotes protective efficacy in non-human primates. Sci Immunol (2021) 6(61):eabh3634. doi: 10.1126/sciimmunol.abh3634 34266981 PMC9119307

[31] Liu Z Xu W Xia S Gu C Wang X Wang Q. RBD-fc-based COVID-19 vaccine candidate induces highly potent SARS-CoV-2 neutralizing antibody response. Signal Transduct Target Ther (2020) 5(1):282. doi: 10.1038/s41392-020-00402-5 33247109 PMC7691975

[32] Jin P Guo X Chen W Ma S Pan H Dai L. Safety and immunogenicity of heterologous boost immunization with an adenovirus type-5-vectored and protein-subunit-based COVID-19 vaccine (Convidecia/ZF2001): A randomized, observer-blinded, placebo-controlled trial. PloS Med (2022) 19(5):e1003953. doi: 10.1371/journal.pmed.1003953 35617368 PMC9187065

[33] Our World in Data. Share of people who received at least one dose of COVID-19 vaccine (2022). Available at: https://ourworldindata.org/grapher/share-people-vaccinated-covid?Country=OWID_WRL~Low+income~ZAF.

[34] He X Hong W Pan X Lu G Wei X. SARS-CoV-2 omicron variant: Characteristics and prevention. MedComm (2021) 2(4):838–45. doi: 10.1002/mco2.110 PMC8693031 34957469

[35] Zang J Zhu Y Zhou Y Gu C Yi Y Wang S. Yeast-produced RBD-based recombinant protein vaccines elicit broadly neutralizing antibodies and durable protective immunity against SARS-CoV-2 infection. Cell Discovery (2021) 7(1):71. doi: 10.1038/s41421-021-00315-9 34408130 PMC8372230

[36] Xu K Gao P Liu S Lu S Lei W Zheng T. Protective prototype-beta and delta-omicron chimeric RBD-dimer vaccines against SARS-CoV-2. Cell (2022) 185(13):2265–78.e14. doi: 10.1016/j.cell.2022.04.029 35568034 PMC9042943

[37] Planas D Veyer D Baidaliuk A Staropoli I Guivel-Benhassine F Rajah MM. Reduced sensitivity of SARS-CoV-2 variant delta to antibody neutralization. Nature (2021) 596(7871):276–80. doi: 10.1038/s41586-021-03777-9 34237773

[38] Liu C Ginn HM Dejnirattisai W Supasa P Wang B Tuekprakhon A. Reduced neutralization of SARS-CoV-2 B.1.617 by vaccine and convalescent serum. Cell (2021) 184(16):4220–4236.e13. doi: 10.1016/j.cell.2021.06.020 34242578 PMC8218332

[39] Wang Y Liu C Zhang C Wang Y Hong Q Xu S. Structural basis for SARS-CoV-2 delta variant recognition of ACE2 receptor and broadly neutralizing antibodies. Nat Commun (2022) 13(1):871. doi: 10.1038/s41467-022-28528-w 35169135 PMC8847413

[40] Gurung AB Ali MA Lee J Farah MA Al-Anazi KM Al-Hemaid F. Structural and functional insights into the major mutations of SARS-CoV-2 spike RBD and its interaction with human ACE2 receptor. J King Saud Univ Sci (2022) 34(2):101773. doi: 10.1016/j.jksus.2021.101773 34955621 PMC8686452

[41] Horiuchi K Kosugi S Abe H Ueda Y. Fulminant myocarditis after the first dose of mRNA-1273 vaccination in a patient with previous COVID-19: a case report. Eur Heart J Case Rep (2022) 6(7):ytac290. doi: 10.1093/ehjcr/ytac290 35860438 PMC9278249

[42] Richmond P Hatchuel L Dong M Ma B Hu B Smolenov I. Safety and immunogenicity of s-trimer (SCB-2019), a protein subunit vaccine candidate for COVID-19 in healthy adults: a phase 1, randomised, double-blind, placebo-controlled trial. Lancet (2021) 397(10275):682–94. doi: 10.1016/S0140-6736(21)00241-5 PMC7906655 33524311

[43] Ellis RD Martin LB Shaffer D Long CA Miura K Fay MP. Phase 1 trial of the plasmodium falciparum blood stage vaccine MSP1(42)-C1/Alhydrogel with and without CPG 7909 in malaria naive adults. PloS One (2010) 5(1):e8787. doi: 10.1371/journal.pone.0008787 20107498 PMC2809736

[44] Ge J Pu X Li J Xia X Wang X Sun J. The combination of aluminum adjuvant and CpG adjuvant can enhance the immunogenicity of recombinant HPV vaccines. J Infect Dis Ther (2022) 10(3):495. doi: 10.4172/2332-0877.1000495

