# Supplementary material for: A vaccine based on the yeast-expressed receptor-binding domain (RBD) elicits broad immune responses against SARS-CoV-2 variants
Source: Front Immunol. 2022 Nov 9;13:1011484. doi: 10.3389/fimmu.2022.1011484 (PMC9682237; doi:10.3389/fimmu.2022.1011484)
Supplement: Supplementary file 1 [file DataSheet_1.docx]

Supplementary Material

## Supplementary Figures


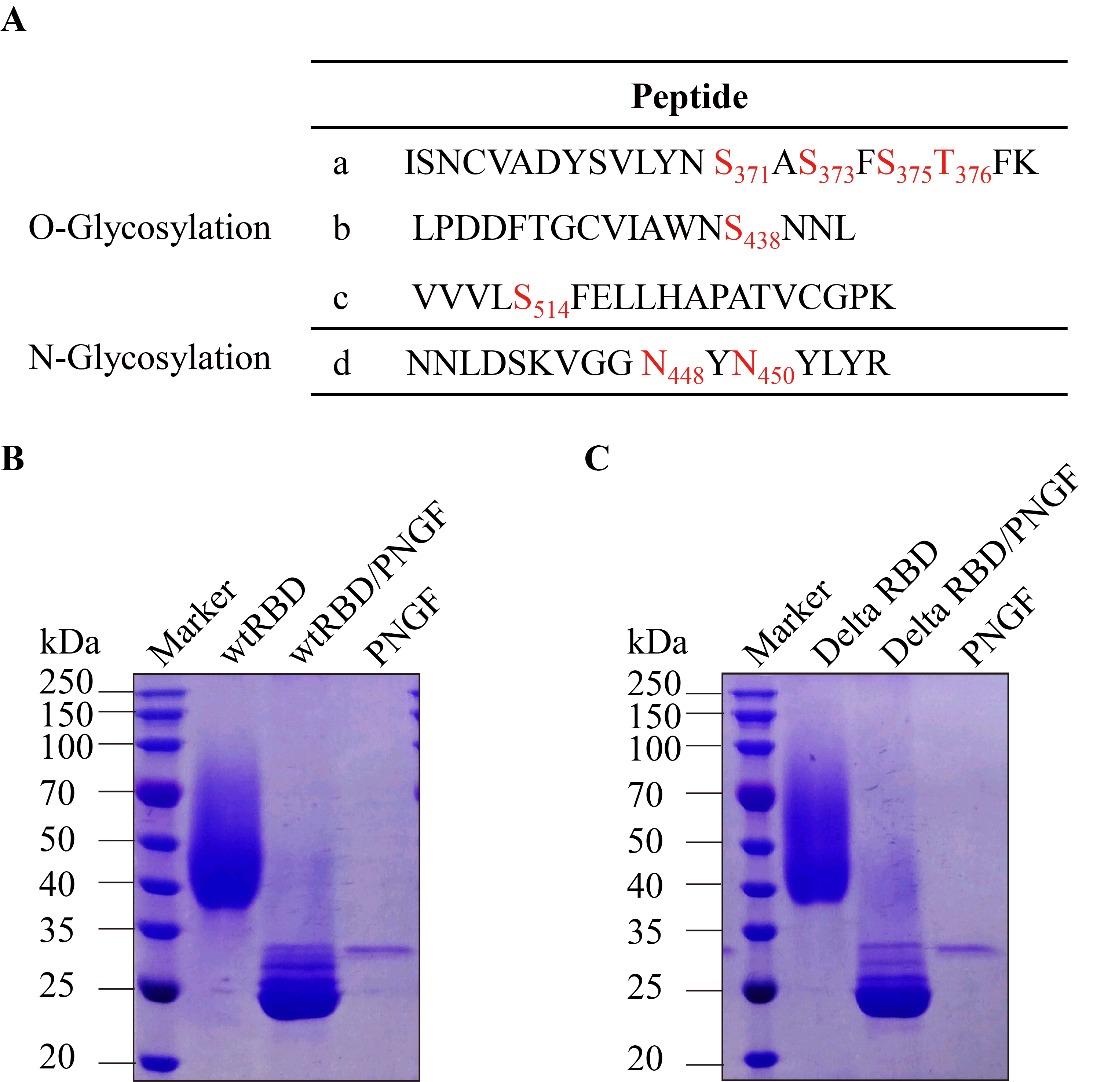


**Supplementary Figure 1.** Glycosylation analysis of the wtRBD and Delta RBD produced in *P. pastoris*. **(A)** Glycosylated sites of wtRBD protein analyzed by liquid chromatography-mass spectrometry (LC-MS). The numbers indicate the amino acids with O-glycosylation (peptides a-c) or N-glycosylation (peptides d). a and b indicate possible O-glycosylation sites; c indicates identified O-glycosylation site; d indicates N-glycosylation sites. **(B and C)** Analysis of glycosylation by PNGase F digestion in wtRBD (B) and Delta RBD (C).


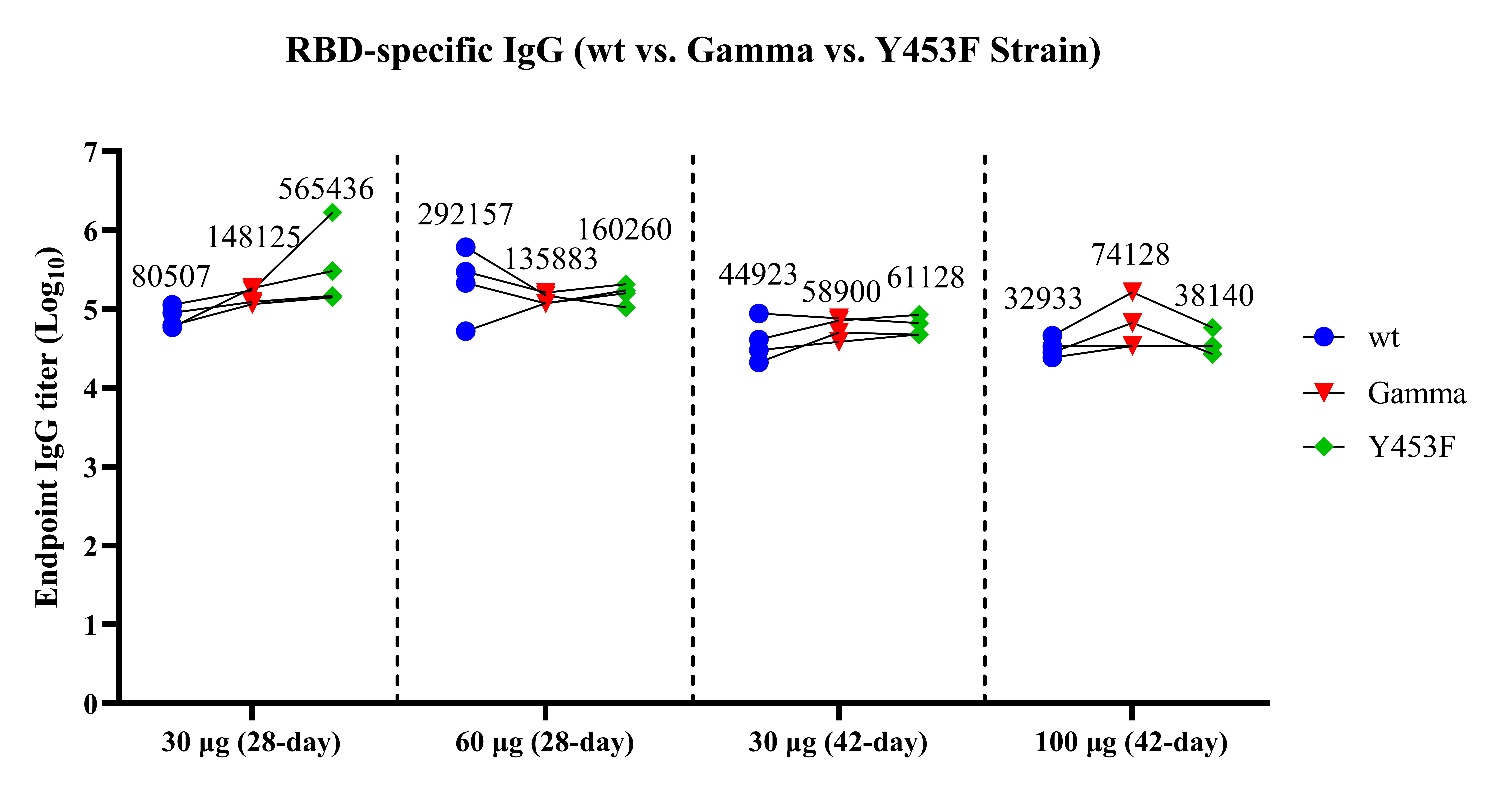


**Supplementary Figure 2.** Cross-reactivity of antisera from mice immunized with wtRBD against Gamma and Mink (Y453F) SARS-CoV-2 variants. The titers of antibody binding to the Gamma and Y453F RBD in serum were measured by ELISA 14 days after the third immunization. Each symbol indicates data from one mouse (n = 4 mice/group); geometric means of each group are shown.


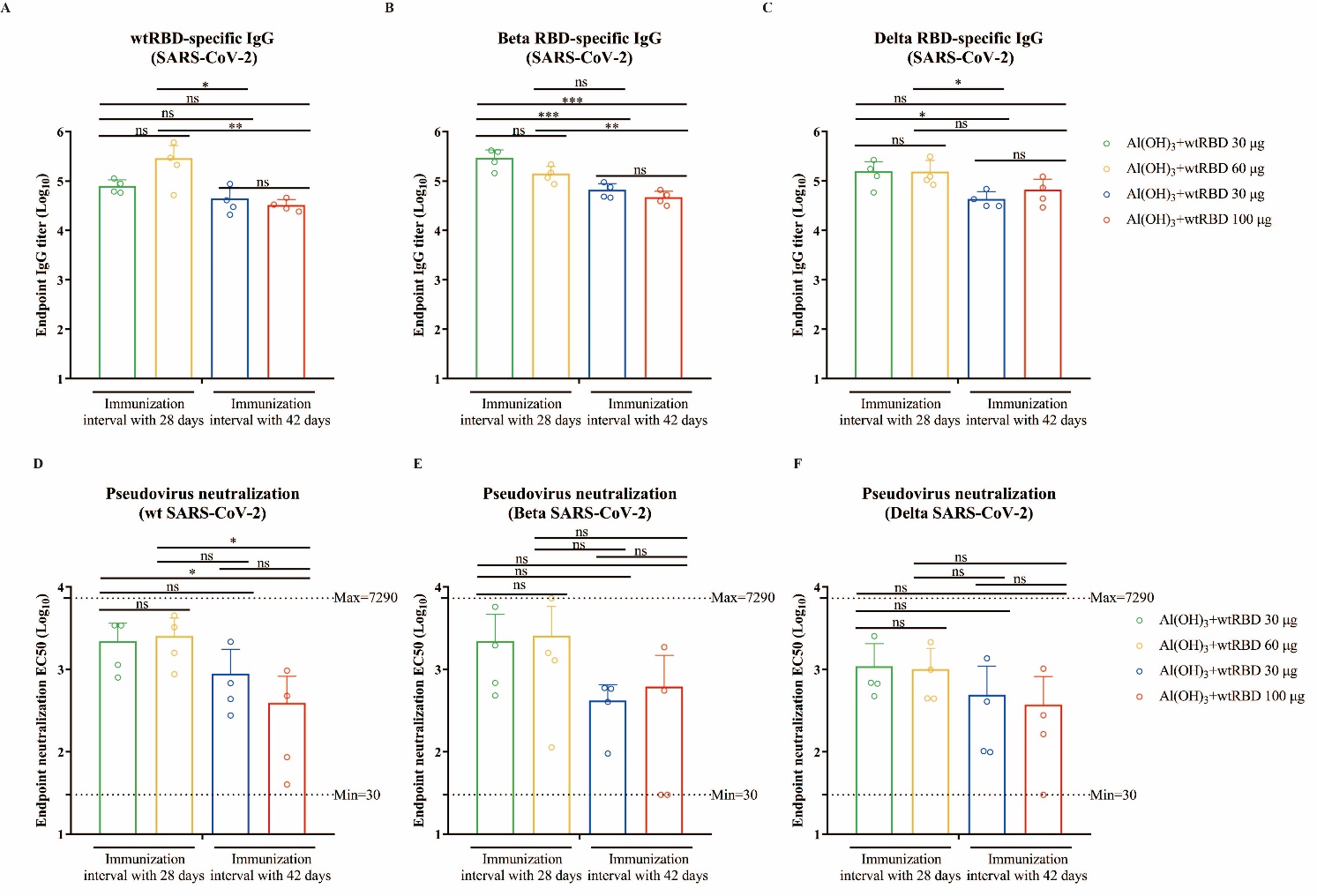


**Supplementary Figure 3.** Higher cross-reactivity of antisera from mice immunized with yeast-expressed wtRBD using immunization program with an interval of 28 days. **(A-C)** Detection of the wild-type (A), Beta (B) or Delta (C) RBD-specific IgG titers in the sera from immunized mice using immunization program with interval of 28 days or 42 days. **(D-F)** The NAb titers against wild-type (D), Beta (E), or Delta (F) SARS-CoV-2 in sera of immunized mice were measured 14 days after the third dose by the pseudovirus neutralization assay. Data are the mean ± SD. *p*-values were calculated using one-way ANOVA. * *p* < 0.05, ** *p* < 0.01, *** *p* < 0.001; ns, not significant.

**
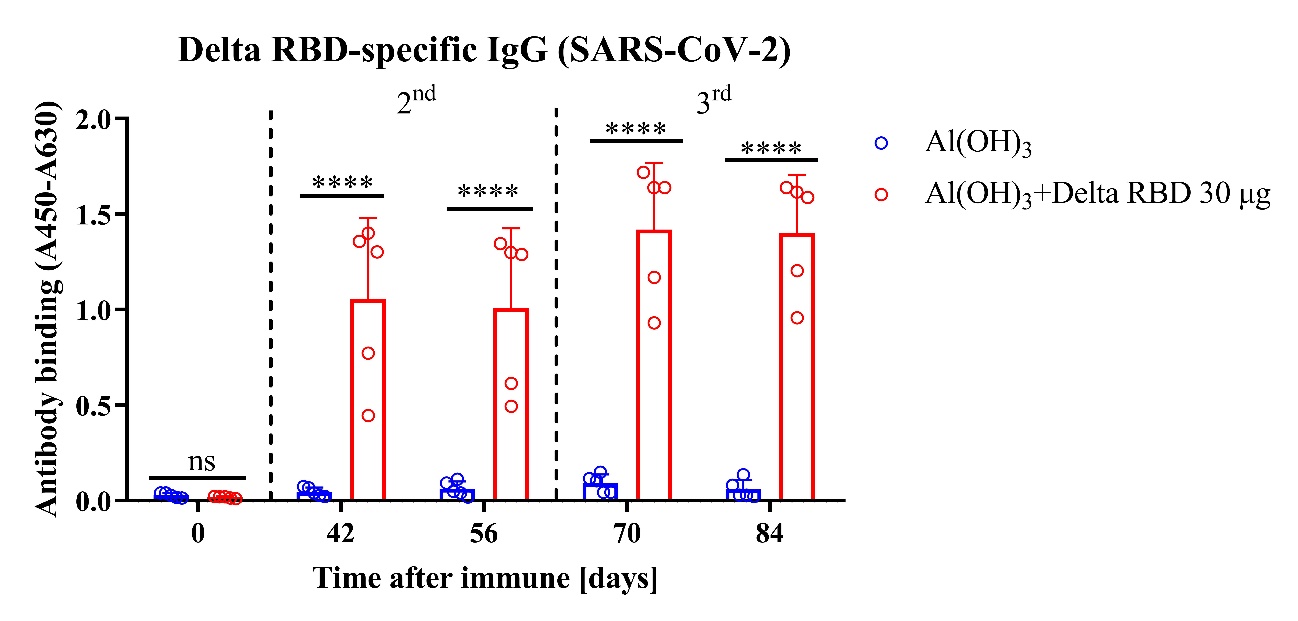
**

**Supplementary Figure 4.** Humoral immune responses elicited by yeast-expressed Delta RBD in mice. Antibodies binding to the Delta RBD in sera from immunized mice with a 28-day interval were measured by ELISA. Data are the mean ± SD. *p*-values were calculated using unpaired Student’s t-test. **** *p* < 0.0001; ns, not significant.

**
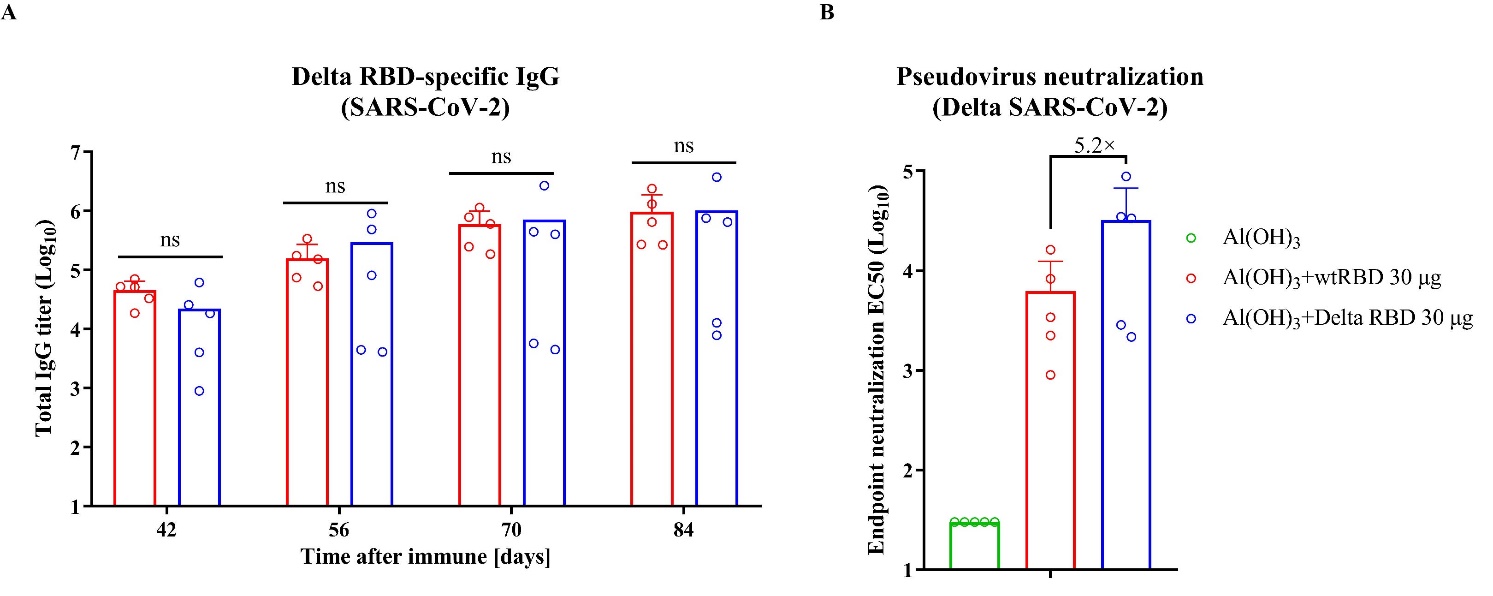
Supplementary Figure 5.** Humoral immune responses against the Delta SARS-CoV-2 variant induced by yeast-expressed wtRBD or Delta RBD in mice. Groups of mice were immunized with 30 μg of wtRBD or Delta RBD in the presence of Alum adjuvant three times with an interval of 28 days apart, and only PBS plus Alum adjuvant as a negative control. Serum samples were obtained at the time points as shown in Figure 4D (Immunization program 3). **(A)** Detection of the Delta RBD-specific IgG titers in sera from immunized mice with wtRBD (Red) or Delta RBD (Blue). **(B)** The NAb titers against the Delta SARS-CoV-2 variant in the endpoint sera (day 84) of immunized mice were measured by the pseudovirus neutralization assay. Data are the mean ± SD. *p*-values were calculated using unpaired Student’s t-test. ns, not significant.

**
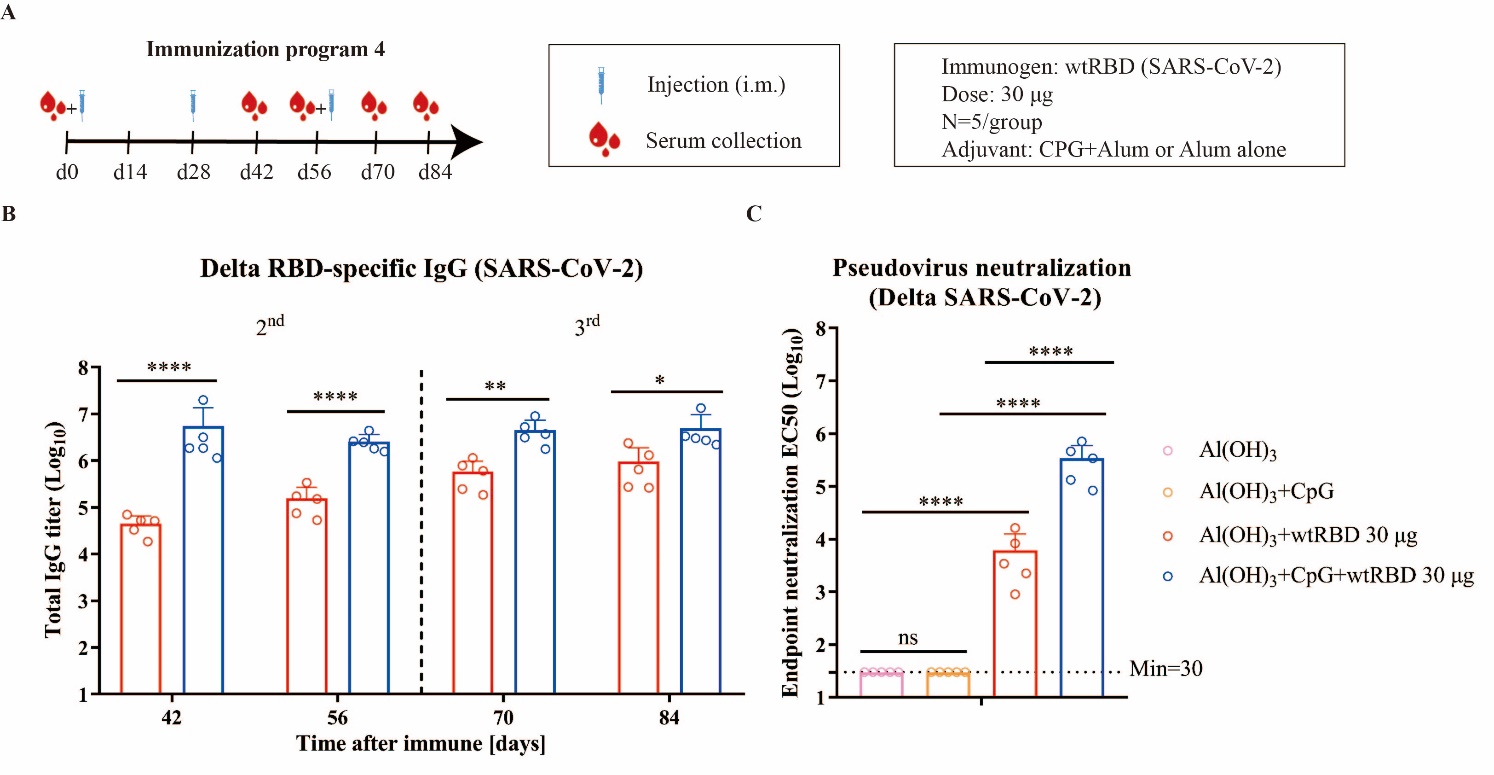
**

**Supplementary Figure 6.** Cross-reactivity of antisera from mice immunized with wtRBD against Delta SARS-CoV-2 variants. **(A)** Schematic of immunization program 4. Groups of mice were immunized with 30 μg of wtRBD in the presence of Alum or CpG+Alum adjuvant three times in 28-day intervals, and only PBS plus adjuvant were used as a negative control. Serum samples were obtained at the indicated time points. **(B)** Detection of the Delta RBD-specific IgG titers in sera from immunized mice at 28-day intervals. **(C)** The NAb titers against the Delta SARS-CoV-2 variant in the endpoint sera (day 84) of immunized mice were measured by the pseudovirus neutralization assay. Data are the mean ± SD. *p*-values were calculated using unpaired Student’s t-test. * *p* < 0.05, ** *p* < 0.01, **** *p* < 0.0001; ns, not significant.


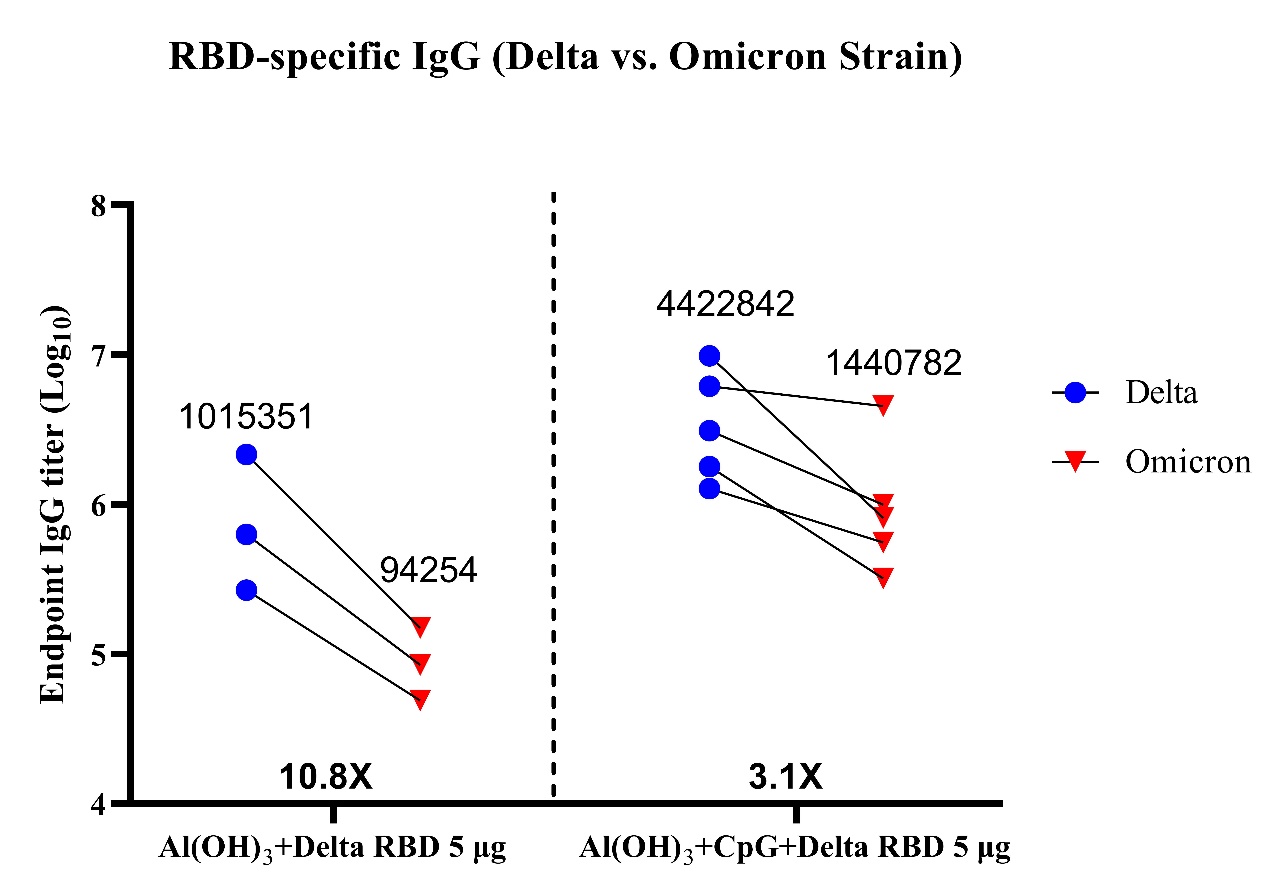


**Supplementary Figure 7.** Cross-reactivity of the antisera from mice immunized with Delta RBD against the Omicron variant. The endpoint (day 84) serum samples from the mice immunized with 5 μg of Delta RBD (Figure 5A) were used for the Omicron RBD-specific IgG binding antibodies. Each symbol indicates data from one mouse; geometric means of each group are shown.


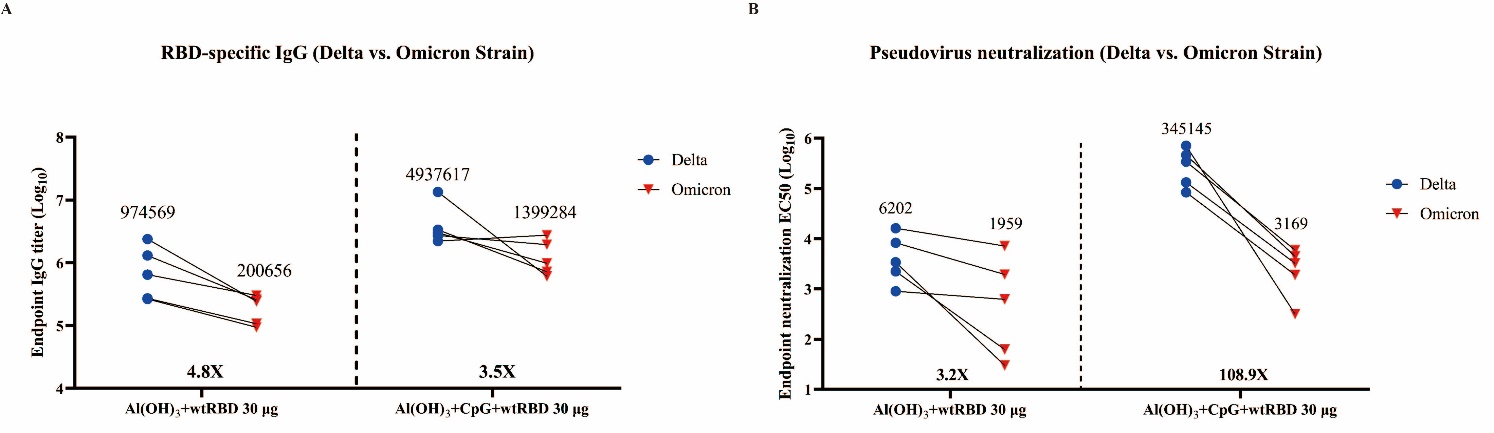


**Supplementary Figure 8.** Cross-reactivity of the antiserum from mice immunized with wtRBD against the Omicron SARS-CoV-2 variants. **(A)** The titers of antibody binding to the Omicron RBD in serum were measured by ELISA at four weeks after the third immunization. **(B)** The NAb titers against Omicron SARS-CoV-2 variants in antiserum were evaluated at 28 days after the third dose by the pseudovirus neutralization assay. Each symbol indicates data from one mouse (n = 5 mice/group); geometric means of each group are shown.


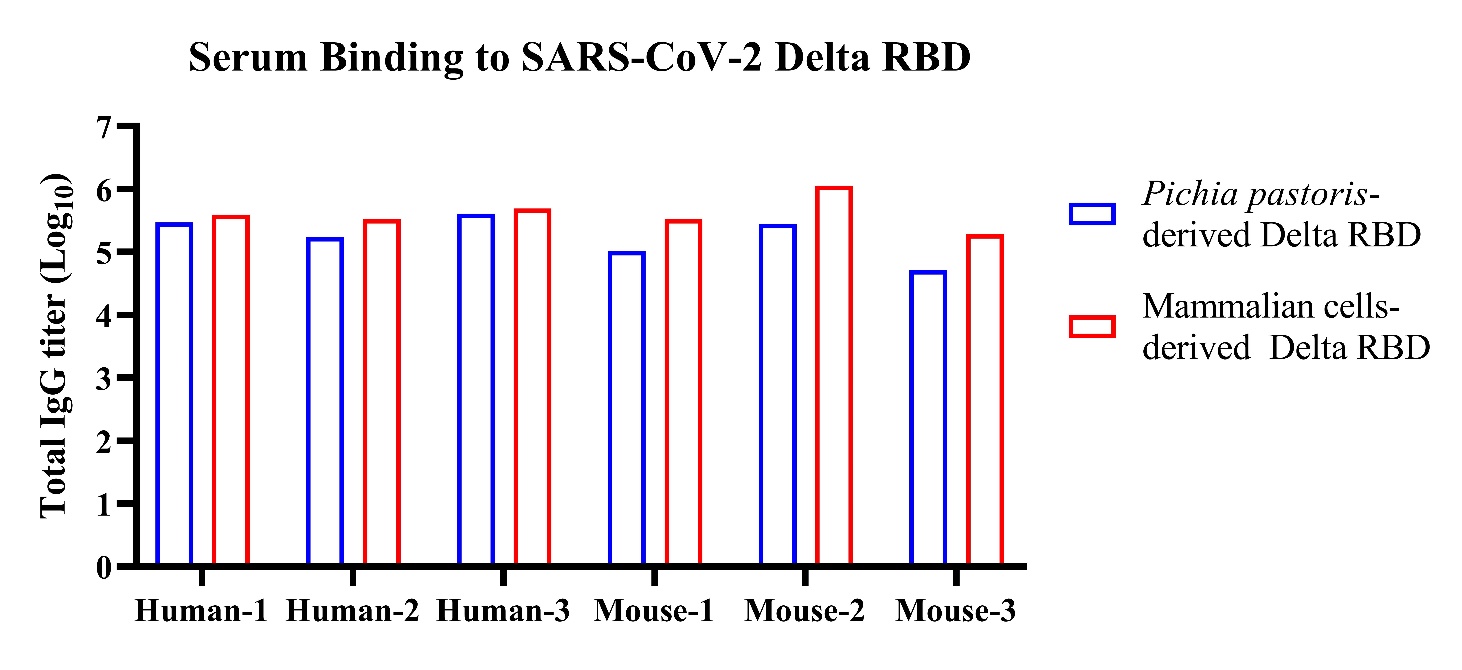


**Supplementary Figure 9.** Comparison of the antigenicity of Delta RBD derived from *Pichia pastoris* and mammalian cells. Human serum samples containing RBD antibody were obtained from the volunteer vaccinated with recombinant RBD protein expressed in CHO cells. Mouse serum samples containing RBD antibody were obtained from the group immunized with 5 μg of Delta RBD protein expressed in *Pichia pastoris* cells (Figure 5A). Delta RBD-specific antibody titers were measured by ELISA. Delta RBD derived from *Pichia pastoris* and mammalian cells were pre-coated in 96-well plates with 200 ng per well, and serial dilutions (3-fold with an initial dilution of 40) of the serum samples were tested by ELISA.


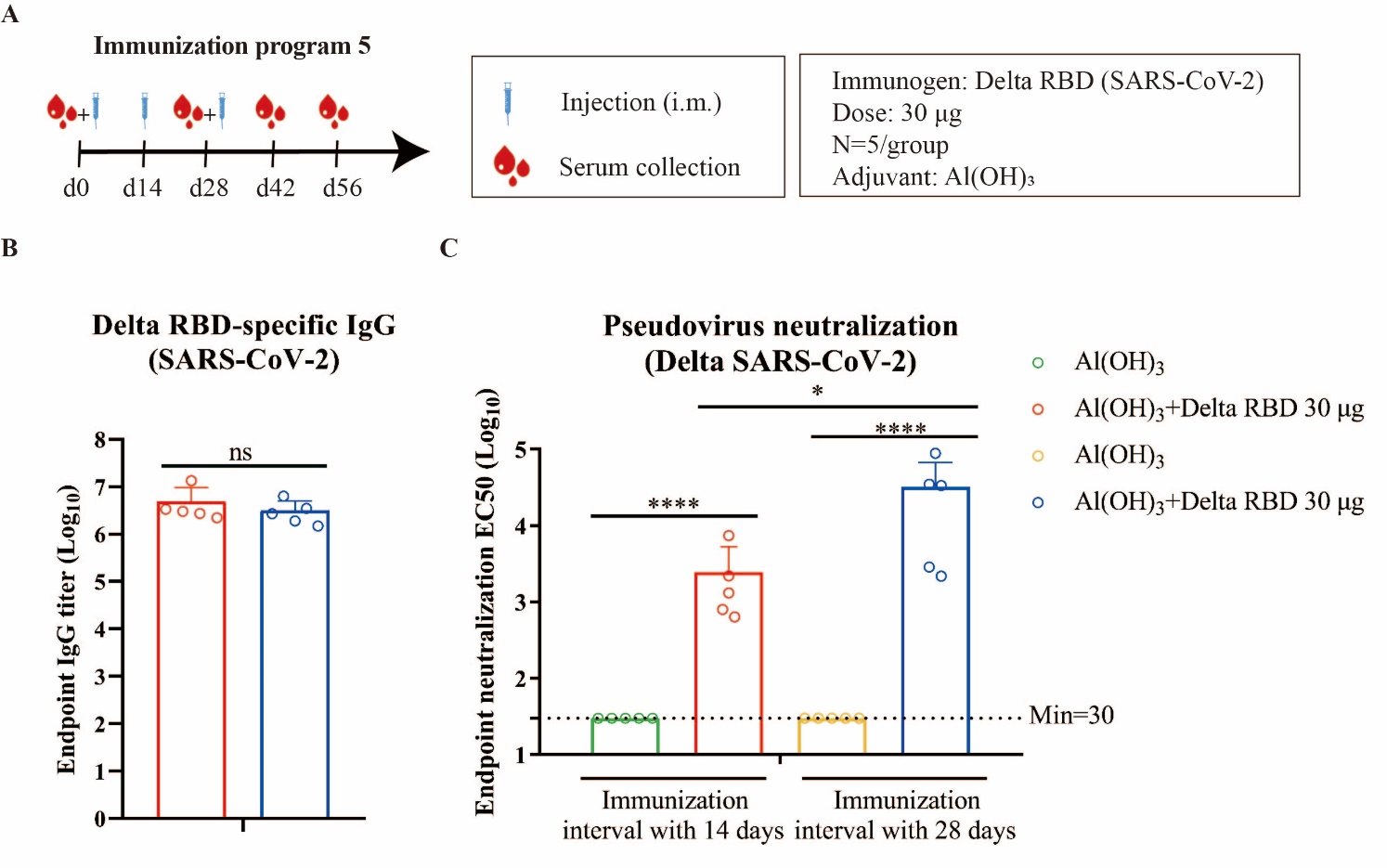


**Supplementary Figure 10.** Cross-reactivity of serum samples from mice injected with yeast-expressed Delta RBD. **(A)** Schematic of immunization program 5. Groups of mice were immunized with 30 μg of wtRBD in the presence of Alum adjuvant three times with a 14-day interval, and only PBS plus adjuvant as a negative control. The endpoint serum samples from the mice immunized with Delta RBD at 28 days after the third dose were used for the IgG binding antibody and NAb assays. **(B)** Detection of the Delta RBD-specific IgG titers in endpoint sera from immunized mice at 14-day (red, immunization program 5) or 28-day (blue, immunization program 3) intervals. Data are the mean ± SD. *p*-values were calculated using an unpaired Student’s t-test. ns, not significant. **(C)** The NAb titers against the Delta SARS-CoV-2 variant in endpoint sera of immunized mice were measured by the pseudovirus neutralization assay. Data are the mean ± SD. *p*-values were calculated using one-way ANOVA. * *p* < 0.05, **** *p* < 0.0001; ns, not significant.

## Supplementary Tables

| **Gene name** | **Sequence (5’-3’)** |
| --- | --- |
| **wtRBD-co** | atgagatttccttcaatttttactgctgttttattcgcagcatcctccgcattagctgctccagtcaacactacaacagaagatgaaacggcacaaattccggctgaagctgtcatcggttactcagatttagaaggggatttcgatgttgctgttttgccattttccaacagcacaaataacgggttattgtttataaatactactattgccagcattgctgctaaagaagaaggggtatctctcgagaaaagagaggctgaagctagagttcagccaactgagtccatcgtcagattcccaaacatcaccaacttgtgcccattcggtgaggttttcaacgctactagattcgcttccgtttacgcctggaacagaaagagaatctccaactgcgttgctgactactccgtcttgtacaactctgcttcattctccaccttcaagtgctacggtgtttccccaactaagttgaacgacctgtgtttcactaacgtctacgccgactccttcgttattagaggtgacgaggttagacagatcgctccaggtcaaactggtaagatcgctgactacaactacaagctgccagacgacttcaccggttgtgttattgcttggaactccaacaacctggactccaaggttggtggtaactacaattaccggtaccgtctgttcagaaagtccaacctgaagccattcgagagagacatctccaccgaaatctaccaagctggttccaagccatgtaacggtgttgagggtttcaactgttacttcccattgcagtcctacggtttccagccaactaatggtgttggttaccagccatacagagtcgtcgttttgtccttcgagttgttgcatgctccagctactgtttgcggtccaaagaagtccactaacctggtcaagaacaagtgcgtcaactttgatgatgacgacaagggtggtggtggttcccatcatcatcatcatcattga |
| **wtRBD-co1** | ATgAGAGTCCAACCGACTGAGTCCATCGTTCGATTTCCCAACATCACTAACCTGTGTCCTTTCGGGGAGGTATTTAATGCGACGCGGTTCGCTTCAGTGTACGCTTGGAATAGGAAACGTATCTCTAATTGTGTCGCAGATTACTCAGTTCTTTACAACAGTGCCAGTTTTTCAACGTTTAAGTGTTATGGCGTGAGCCCGACAAAGCTGAACGATCTATGTTTTACTAACGTATATGCAGACAGCTTCGTTATACGTGGGGATGAAGTCCGCCAAATTGCCCCTGGGCAAACCGGAAAAATTGCCGACTATAACTATAAGTTGCCGGACGATTTTACCGGCTGCGTGATAGCATGGAACTCGAACAATTTAGACAGCAAGGTAGGCGGAAATTACAATTACCTATATCGACTTTTCCGGAAGTCGAATCTCAAACCATTTGAACGCGACATAAGTACGGAGATTTACCAGGCGGGGTCCACACCATGCAACGGAGTTGAAGGCTTCAATTGTTATTTCCCTCTGCAGTCTTACGGTTTTCAGCCCACTAATGGAGTAGGTTATCAACCCTACAGGGTGGTTGTGTTATCGTTCGAATTGCTCCACGCTCCCGCGACAGTATGCGGTCCAAAAAAGTCCACCAACCTAGTCAAAAACAAATGCGTCAATTTCGATGATGACGACAAGGGTGGTGGTGGTTCCCATCATCATCATCATCATTAA |

Blue, α-factor; Pink, RBD; Orange, EK; Green, Linker; Purple, 6×His.

**Table S1.** The codon-optimized sequences of SARS-CoV-2 RBD.

| **Number** | **Primer name** | **Primer sequence (5’-3’)** |
| --- | --- | --- |
| 1 | afactor-F1-EcoRI | aCCgaattcatgagatttccttcaatttttac |
| 2 | afactor-R1-EcoRI | agcttcagcctctcttttctcgagag |
| 3 | RBDco1-F2 | tctcgagaaaagagaggctgaagctagagtccaaccgactgagtccatc |
| 4 | RBD-SalI-R | attgtcgacttaatgatgatgatgatgatgggaaccaccaccacc |
| 5 | Delta RBD-R1 | tgtcgcgttcaaatggtttgagattcgacttccggaaaagtcgatatCggtaattgtaa |
| 6 | Delta RBD-F2 | aaccatttgaacgcgacataagtacggagatttaccaggcggggtccaAaccatgcaac |
| 7 | 5'AOX | GACTGGTTCCAATTGACAAGC |
| 8 | 3'AOX | GCAAATGGCATTCTGACATCC |

**Table S2.** The primers utilized for the constructed plasmids.
